# Effects of Tai Chi and Qigong on cognitive and physical functions in older adults: systematic review, meta-analysis, and meta-regression of randomized clinical trials

**DOI:** 10.1186/s12877-023-04070-2

**Published:** 2023-06-06

**Authors:** Moonkyoung Park, Rhayun Song, Kyoungok Ju, Jacqueline C. Shin, Jisu Seo, Xing Fan, Xianqi Gao, Ahyun Ryu, Yuelin Li

**Affiliations:** 1 Chungnam National University, College of Nursing, Daejeon, 35015 Republic of Korea; 2 Indiana State University, Department of Psychology, Terre Haute, IN 47802 USA

**Keywords:** Tai Chi and Qigong, Cognitive function, Physical function, Older adults, Meta-analysis, Meta-regression

## Abstract

**Background:**

Older adults experience age-related declines in physical and cognitive functions due to interactions between aging and chronic diseases. Tai Chi and Qigong (TCQ) might be beneficial in improving the physical function and delaying the cognitive decline of this population. The potential underlying mechanism was explored to determine the effects of TCQ on cognitive function via direct or indirect pathways.

**Purpose:**

The objective of this systematic review was to determine the effects of TCQ on cognitive and physical functions in older adults using meta-analysis, and to determine the impact of TCQ on cognitive function while controlling for physical function using a meta-regression approach.

**Methods:**

A systematic search of 13 electronic databases (in English, Korean, and Chinese languages) identified 10,292 potentially eligible studies published between inception and May 2022. The bias in individual studies was assessed using the Cochrane Risk of Bias (version 2.0) tool. The heterogeneity of the studies was evaluated using a 95% prediction interval, and the meta-analysis and meta-regression were implemented using the Comprehensive Meta-Analysis (version 3) software.

**Results:**

Our search identified 17 randomized studies (*n* = 2,365, mean age = 70.3 years). The results of the meta-analysis that used a random-effects model indicated that TCQ had significant effects on both cognitive (Hedges' g = 0.29, 95% confidence interval [CI] = 0.17 to 0.42) and physical (Hedges' g = 0.32, 95% CI = 0.19 to 0.44) functions. We used meta-regression to explore the effect size of TCQ in association with physical function level. The regression model was significant (Q = 25.01, *p* = .070), and 55% of the heterogeneity was explained by physical function as a moderator variable. The effects of TCQ on cognitive function remained significant in this model when controlling for the effect of physical function (β = 0.46, *p* = .011).

**Conclusion:**

This meta-regression of 17 randomized studies strongly suggests that TCQ has beneficial effects on physical and cognitive functions in older adults. The effect of TCQ on cognitive function remained significant after taking into account the significant effects of physical function as a moderator. The findings imply the potential health benefits of TCQ by promoting cognitive function in older adults directly and indirectly through enhancing physical function.

**PROSPERO registration number:**

*PROSPERO international prospective register of systematic reviews, registration ID CRD42023394358.

**Supplementary Information:**

The online version contains supplementary material available at 10.1186/s12877-023-04070-2.

## Background

Aging is the progressive accumulation of time-related changes, and age-related cognitive decline has become a global public health problem [1, 2]. Older adults remember less and walk more slowly than younger adults, which is associated with generalized impairments of physical and cognitive functions [3]. As people age, some experience age-related declines in physical and cognitive functioning from interactions between aging and disease [4], which increases their risks of dependence and premature death [5].

Tai Chi and Qigong (TCQ) is a popular mind–body intervention that shares the core features of Chinese martial arts and meditative movements through smooth and continuous body movements and breathing [6, 7]. There are many similarities between Tai Chi and Qigong in how they focus on the body (posture and movement), breath, and mind (meditation and mindfulness) [8, 9]. The mind–body exercise component of TCQ is a potent lifestyle factor playing a critical role in preserving and promoting healthy cognitive aging [8, 9]. Therefore, Tai Chi and Qigong were considered equivalent interventions and were grouped together in the present review.

A key neurotrophin that influences cognitive function is brain-derived neurotrophic factors (BDNFs). In the cerebral cortex and hippocampus, BDNFs are shown to facilitate neurogenesis and promote synaptic plasticity [10]. Exercise has the potential to directly or indirectly affect the synthesis and release of hippocampal BDNFs’ production [11, 12]. TCQ as an alternative aerobic exercise might be beneficial for delaying cognitive decline in older adults [13], possibly via the upregulation of BDNFs [14]. A randomized controlled trial (RCT) involving people with mild dementia found that a TCQ program might improve cognitive function and mental well-being in this population [15]. A previous meta-analysis indicated that TCQ might improve the cognitive function of middle-aged and older adults with mild cognitive impairment (MCI) [16].

TCQ could affect neural processes and cognitive performance via various pathways, such as aerobic exercises, by engaging learning processes through meditative aspects and from a potential mechanism underlined in East Asian traditions. First, TCQ comprises mild-to-moderate exercise levels depending on specific features [17–19] that are known to have clear cognitive benefits [20, 21]. Aerobic exercise has been found to induce changes in neurotransmitters, neural growth factors, and functional network properties that strengthen neuroplasticity [22, 23] and improve brain perfusion [24]. Consistent with these expectations, several systematic reviews of Tai Chi have shown structural [25, 26] and functional [25, 27, 28] changes as a result of neuroimaging techniques, including in cortical thickness, functional connectivity, and homogeneity of the brain, and executive network neural function [21]. Previous intervention studies also demonstrated that TCQ improved cognitive functioning by changing neural activity [28, 29]. A randomized trial reported that 15 min of Qigong training increased EEG alpha activity, leading to a relaxed state of mind, and theta activity, resulting in internalized attention [28]. Compared to general aerobic exercise, Tai Chi exercise improved cognitive flexibility among healthy adults by affecting brain functional specialization [29].

Second, because TCQ requires practitioners to learn and perform highly coordinated sequences of whole-body postures and movements, cognitive and neural processes involved in attention, sequence learning, and body coordination are heavily involved. TCQ may enhance the neural mechanisms activated during the learning and practice of those exercises and enhance the learning of other sequencing and body coordination activities controlled by the same brain areas. Indeed, learning TCQ enhances growth-factor levels, such as those of BDNFs [22, 30], which in turn enhances learning [22, 31]. TCQ may strengthen specialized areas of the brain responsible for declarative and procedural learning that are utilized when learning the action sequences [32, 33].

Third, the meditative state is a central and unique aspect of mind–body exercises such as TCQ. Meditation improves attention and executive functions [34, 35]. The meditative aspect of TCQ would be expected to benefit older adults since meditation is associated with cognitive benefits in those with cognitive decline [36, 37] and structural brain changes in neurodegenerative disease [38]. The calm mental-focus characteristic of TCQ is conducive to reducing stress, which negatively affects memory in both Tai Chi [39, 40] and Qigong [28, 41]. The meditative aspect of TCQ may affect cognition by enhancing mood [42, 43], which influences perception, attention, and memory [44, 45]. The mindfulness and mental focus characteristics in TCQ would benefit cognition beyond what would be expected in other exercises with similar intensity.

Finally, TCQ was developed based on Taoist philosophy and Traditional Chinese Medicine (TCM) and was designed to stimulate the flow of qi—a form of physical and mental energy—in the internal organs, musculature, and system of qi pathways, or *meridians*, and thereby improve physiological function [46]. Together, meditation and movements are postulated to stimulate acupuncture points and affect the qi and the efficiency of qi flow through the meridians [47]. Both Taoist theories about 'life-nurturing' and TCM postulate that strengthening the qi leads to mental and spiritual clarity as well as improved body health and well-being [48, 49]. However, the potential association between the cultivation and enhanced circulation of qi and cognitive benefits requires further exploration.

Recent empirical investigations of the benefits of TCQ have primarily focused on physical functions such as maintaining physical aerobic endurance, lower body strength, balance, mobility, gait speed [50, 51], and motor performance [52]. However, exercise-induced benefits on physical and cognitive functions are generally interrelated. For example, previous cross-sectional studies found that older adults with better physical function, frequently indicated by the stronger grip and faster gait, may present better cognitive performance in executive function, memory, and processing speed [53–58]. In addition, a previous meta-analysis also supported the associations between cognitive and physical functions and the impact of exercise training, indicating that exercise-induced improvements in physical function are often accompanied by improvements in cognitive function [59].

As a mind–body exercise that is safely applicable to older adults with impaired physical and cognitive functions, TCQ has been found to be beneficial for delaying impairments in these functions in older adults [60]. Several meta-analysis studies have reported that TCQ is effective in improving cognitive functions [16, 61] and physical functions [62], but no studies have focused on the relationship between physical and cognitive functions. Based on the close association between physical and cognitive functions, we hypothesized that the effects of TCQ on cognitive function could be induced both directly and indirectly via physical functions. While there is promising evidence for cognitive improvement from practicing TCQ, the potential mechanisms underlying the effect of TCQ on cognitive function via indirect or direct pathways have yet to be explored.

Meta-regression involves the integration of meta-analysis and linear regression and aims to explain heterogeneity at the study level rather than at the individual level [63]. Meta-regression can identify multiple covariates, including categorical and continuous variables, and both linear and nonlinear relationships with effect sizes can be assessed [64]. The present systematic review therefore aimed to use meta-regression to determine the effectiveness of TCQ in improving cognitive and physical functions in older adults and to determine if the effect of TCQ on cognitive function remains significant after controlling for physical function.

## Methods

### Search strategy

The electronic literature published up to May 2022 was searched: six English literature databases (PubMed, Embase, Cochrane Library, CINAHL, ProQuest, and OVID), seven Korean databases (Research Information Sharing Service [RISS], Korean Studies Information Service System [KISS], National Digital Science Library, DBpia, Korea Scholar, National Assembly Library, and Korean Citation Index), and four Chinese databases (Wanfang, China Science and Technology Journal Database [VIP], and Chinese National Knowledge Infrastructure [CNKI]). Three search term groups (Tai Chi, clinical trials, and cognition) were used to find studies that yielded results on both physical and cognitive functions. These search terms were used in combination with their Medical Subject Headings (MeSH) terms, keywords, and synonyms in the search.

The main search terms applied to the Chinese databases were CNKI terms and simplified Chinese characters: “Taichi (太极)” OR “Qigong (气功)” OR “Taiji Quan(太极拳)” OR “Baduan jin (八段锦)”. All articles extracted from each database were screened for duplicates and organized using EndNote (version 20) reference management software. The following characteristics of the included studies were summarized in a Microsoft Excel spreadsheet: publication year, language, population, intervention types, outcome measurements, and comparison groups.

### Eligibility criteria

Only RCTs were included in our analysis. The eligibility criteria included studies in which (1) participants are older adults (≥ 60 years) who lived in community settings (i.e., nursing homes, assisted living facilities, or their own homes), (2) participants did not have any disease that affected cognitive function or only had MCI, (3) the intervention included Tai Chi or Qigong, (4) the article included results on both physical (ADL, balance, walking ability, muscle strength, flexibility, and physical health) and cognitive functions (cognitive performance, attention/executive function, memory, and linguistic competence), and (5) the article was published in English, Korean, or Chinese. We excluded articles in which participants were hospitalized or residing in a rehabilitation facility or had neurodegenerative disorders, such as cancer, dementia, Parkinson’s disease, or Alzheimer’s disease. We considered studies that included control groups participating in alternative exercises, usual care, or no treatment.

#### Data extraction and synthesis

The data that we extracted from the studies were general information (authors, publication year, language), basic characteristics (sample size, intervention type: Tai Chi or Qigong), and intervention length and duration (duration of one session or frequency of sessions). The primary outcome for this review was cognitive function (i.e., global cognitive function, memory and learning, visuospatial ability, and executive function), control condition (active or inactive), and physical function. Outcome data (e.g., means and standard deviations of raw data) were extracted and summarized to evaluate the effects of TCQ on cognitive and physical function.

### Quality and risk-of-bias assessments

The quality and risk of bias for all included articles were assessed using the Cochrane’ Risk of Bias (version 2.0) tool (RoB 2.0) [65]. The Cochrane RoB 2.0 contains five domains of bias: bias arising from the randomization process, bias due to deviations from intended interventions, bias due to missing outcome data, bias in the outcome measurements, and bias in the selection of the reported result. An algorithm judged the risk of bias that arose from each domain, leading to a final decision on ‘low risk of bias,’ ‘some concerns,’ or ‘high risk of bias’ [65]. All authors independently assessed the quality and risk of bias of all articles included in the final review. After dividing the authors into two groups, the outcomes of the review by each group’ were compared. Any disagreements between two independent reviewers were resolved by the reviewers or, if necessary, a third expert reviewer to obtain a consensus.

### Data analysis

Comprehensive Meta-Analysis (CMA V3.0, Biostat, USA) was used to combine effect sizes and assess heterogeneity and publication bias. The random-effects model was employed for the analysis to allow its results to be generalized to comparable studies [66]. Continuous variables were estimated using Hedges’s g effect sizes with 95% confidence intervals (CIs). The absolute heterogeneity level among the effect estimates was assessed by calculating the between-studies standard deviation and 95% prediction interval (PI). The 95% PIs described the expected range of true effects if a new study was conducted. As the between-studies standard deviation and lower and upper limits of the 95% PI were in the same metric as the effect estimates, they offer a reasonable quantification of the heterogeneity level [63, 67–69]. A subgroup analysis was conducted to compare the effects of TCQ according to the intervention duration: short-term (≤ 12 weeks) vs. long-term (> 12 weeks). The moderators of interest included physical function, which was judged to have an influence on the effect size based on an outcome measure of cognitive function. We conducted meta-regression analyses to investigate the potential moderators of the effects of Tai Chi on cognitive function with physical functioning as a covariate. The publication bias was analyzed by constructing funnel plots to explore the possibility of publication bias resulting from the preferential publication of prevalence reports with positive findings and amongst small studies that estimated high prevalence rates [70]. Egger’s regression test was used to evaluate the relationship between the effect size and the standard error [71] to determine the significance of asymmetry.

## Results

### Study selection

A literature search was performed from inception through May 2022 in accordance with the PRISMA 2020 guidelines [72]. Of the 10,292 citations identified in the databases, 2,820 were from English databases (Embase, PubMed, OVID, ProQuest, and CINAHL), 7,154 from Korean databases (KISS, RISS, DBpia, Korea Scholar, and National Assembly Library), and 318 from Chinese databases (Wanfang, VIP, and CNKI). Of the 646 citations identified in the registers, 627 were from CENTRAL and 19 from ClinicalTrials. Manual searches identified two additional citations.

**Fig. 1:**
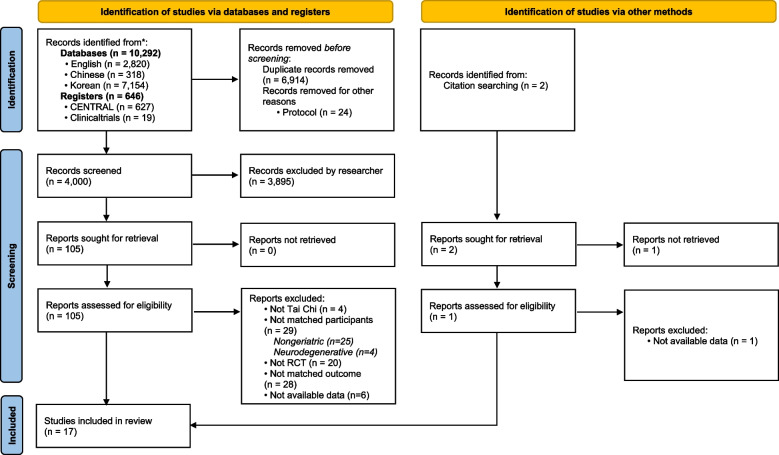
Flow diagram of the study selection process

Duplicate documents were removed using EndNote, and 105 studies were selected according to the inclusion criteria by reviewing titles and abstracts. These 105 studies were screened to exclude non-geriatric (k = 25) or neurodegenerative (e.g. Alzheimer’s disease, Parkinson’s disease, or stroke survivors) studies (k = 4), non-RCTs (k = 20), non-Tai-Chi intervention studies (k = 5), studies that did not evaluate cognitive or physical function as outcomes (k = 28), studies for which data were not available (k = 6), and studies with duplicate participants (k = 1), leaving 17 studies for inclusion in the final analysis. Among the 17 RCTs, one study had two comparison groups (the usual-care and active-control groups); the data of each comparison group were separately entered into the analysis. Two additional studies were found through manual searches, but both were subsequently excluded due to a lack of specific data or not matching the eligibility of the present study. In summary, the qualitative synthesis and quantitative analysis were conducted on 17 studies (Fig. 1).

### Quality of studies and risk of bias

**Fig. 2:**
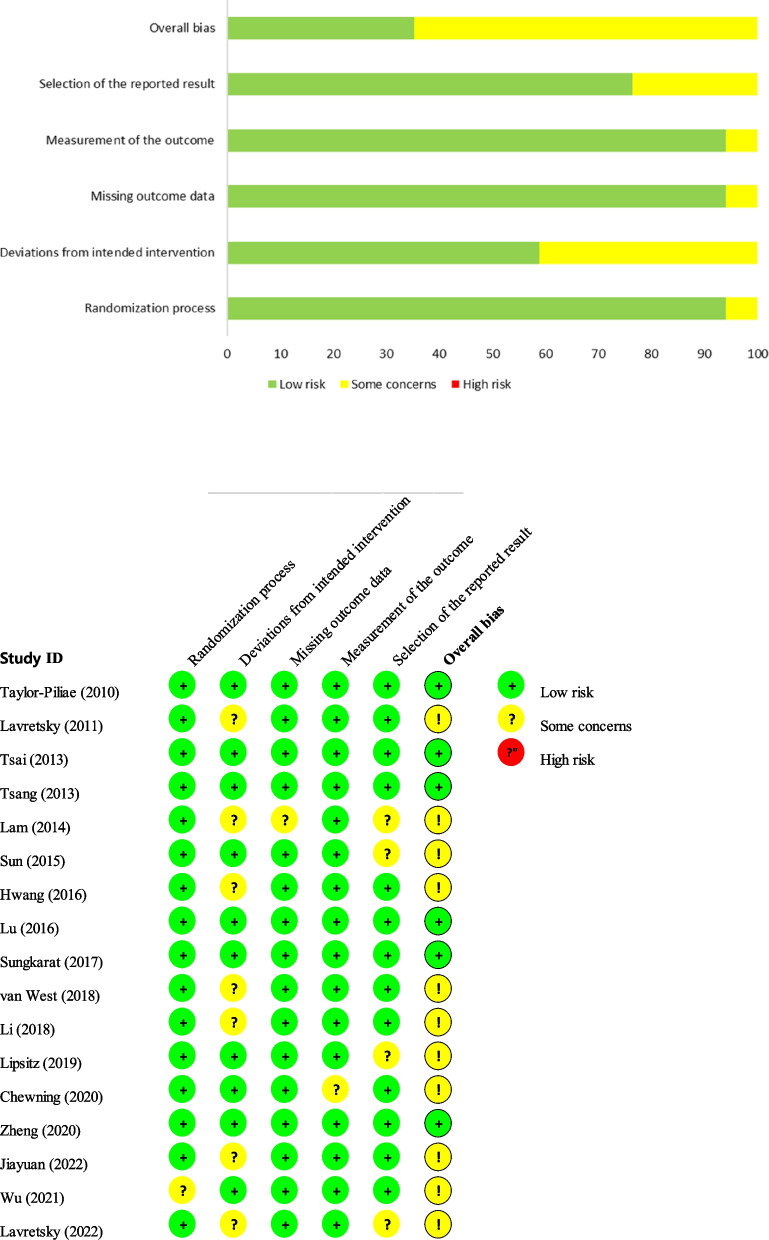
Assessment of risk of bias in the included studies

The quality assessment of the 17 included studies revealed that 6 were low risk (35%) and 11 had some concerns (65%); none were considered high risk. When analyzed by domain, 16 studies (94%) were low risk, and 1 (6%) was considered to have some concerns in the domain that presented the bias, according to the ‘randomization process.’ For the ‘deviation from intended intervention’ domain, there were 10 low-risk studies (58.8%) and 7 with some concerns (41.2%). The risks of bias in the ‘missing outcome data’ and ‘measurement of the outcome’ domains were low in 16 studies (94.1%) and uncertain in 1 (5.9%). Among ‘the selection of the reported result’ domain, there were 13 low-risk studies (76.5%) and 4 (23.5%) with some concerns (Fig. 2).

### Modality and medium

The 17 included RCTs involved 2,235 participants, with 20–456 in each trial, and their mean age was 61–84 years (overall mean age = 70.9 years and 71.8% females). The main characteristics of the studies are listed in Table 1. The subjects included older adults who were healthy (k = 6), prefrail or frail (k = 2), with MCI or amnestic MCI (k = 5), with geriatric depression (k = 2), and who had a risk of falls (k = 2). The intervention was Tai Chi (k = 14) or Qigong (k = 3) applied for a mean duration of 19.7 weeks (range = 4–52 weeks). The control groups received either an alternative intervention (k = 13, health education or alternative exercise) or no treatment (k = 4, usual care or no treatment). All but one study was reported in English (k = 16), with the remaining report written in Chinese. Most intervention settings (k = 12) for older adults were in the community. The non-community settings were described as an elderly service unit from a hospital (k = 1), and a residential home or housing facility (k = 2), or the intervention setting was not reported (k = 2).

**Table 1:** Characteristics of the included studies

Study author, year (Country/Language)	Population				Intervention			Comparison	Outcome measurement		Safety monitoring
	Disease-related characteristics	E (M:F)	C (M:F)	Age, years (group)^b^	TCQ type	Intensity (time × session/W × duration)	Setting		Physical function	Cognitive function	
Taylor-Piliae, 2010 [40] (USA/English)^a^	Age ≥ 60 years, healthy	37 (13:24)	AC: 39 (11:28) UC: 56 (16:40)	70.6 ± 5.9 (E) 68.5 ± 5.0 (AC) 68.2 ± 6.2 (UC)	Tai Chi, 12-form Yang style	1) Adoption phase: 1 h × 5/W (group × 2 + home × 3) × 12 Ws 2) Maintenance phase: 1 h × 4/W(group × 1 + home × 3) × 12 Ws	Community	1) Western exercise 2) Healthy-aging education	OLS FRT Arm curls Chair-stand test Back-scratch test Sit-and-reach test	Animal-naming test Digit span forward Digit span backward	NI
Lavretsky, 2011 [73] (USA/English)	Age ≥ 60 years, geriatric depression	36 (13:23)	37 (15:22)	69.1 ± 7.0 (E) 72.0 ± 7.4 (C)	Tai Chi	Group: 2 h × 1/W × 10 Ws	Community	Health education	SF-36 (physical health)	MMSE CVLT TMT B–A	No adverse events
Tsai, 2013 [74] (USA/English)	Age ≥ 60 years, mild-to-moderate CI with knee OA	28 (6:22)	27 (9:18)	78.9 ± 6.9 (E) 78.9 ± 8.3 (C)	Tai Chi, 12-form Sun style	Group: 20–40 min × 3/W × 20 W	Community	Health education, culture-related activities, and social activities	WOMAC (physical functioning) TUG test Sit-to-stand test	MMSE	No adverse events
Tsang, 2013 [75] (Hong Kong/English)	Age ≥ 60 years, frail	61 (14:47)	55 (15:40)	83.3 ± 6.3 (E) 84.9 ± 6.0 (C)	Novel health Qigong	Group: 1 h × 2/W × 12 Ws	Elderly service unit at hospital	Newspaper reading	Handgrip strength TUG test	LOTCA-G	No adverse events
Lam, 2014 [76] (Hong Kong/English)	Age ≥ 65 years, MCI or amnestic MCI	171 (46:125)	218 (46:172)	77.2 ± 6.3 (E) 78.3 ± 6.6 (C)	Tai Chi, 24 style	1) Adoption phase: group, 30 min × 3/W × 4–6Ws 2) Maintenance phase: home, 30 min × 3/W × 46–48 Ws, refresher lessons every month	Residential home	Stretching and relaxation exercises	BBS	ADA MMSE Digit span backward Visual span backward Delay recall Verbal fluency	NI
Sun, 2015 [77] (China/English)	Age ≥ 60 years, healthy	72 (14:48)	66 (20:46)	68.3 ± 5.9 © 70.1 ± 5.7 (C)	Tai Chi, 24-form Yang style	Group: 1 h × 2/W × 6 Ms	Community	Nonathletic care	OLS 5-m high walking speed 10-m normal walking speed Handgrip strength (both)	MMSE	NI
Hwang, 2016 [78] (Taiwan/English)	Age ≥ 60 years, fall risk	228 (75:153)	228 (77:151)	72.0 ± 8.1 (E) 72.7 ± 8.1 (C)	Tai Chi, 18-form Yang style	Individual: 1 h × 1/W × 6 Ms, self-practice every day	Community	Lower extremity training	Handgrip strength (right hand) Tinetti balance test Tinetti gait test	MMSE	No adverse events
Lu, 2016 [79] (Hong Kong/English)	Healthy female older adults	15 (0:15)	16 (0:16)	72.8 ± 6.7 (E) 67.3 ± 6.6 (C)	Tai Chi, 12-form Yang style	Group: 90 min × 3/W × 16 Ws	Community	Usual care	Computerized gait analysis	Auditory Stroop test: single/dual task	No adverse events
Sungkarat, 2017 [21] (Thailand/English)	Age ≥ 60 years, MCI	33 (2:31)	33 (7:26)	68.3 ± 6.7 (E) 67.5 ± 7.3 (C)	Tai Chi, 10-form style	1) Group: 50 min × 3/W × 3 Ws 2) Home: 50 min × 3/W × 12 Ws	Community	Educational material	PPA	Wechsler Memory Scale; delayed recall Block-design test Digit span forward/backward TMT B–A	No adverse events
van West, 2018 [80] (New Zealand/English)	Age ≥ 60 years, healthy	43 (11:32)	36 (7:29)	69.1 [60-86] (E) 67.6 [60-80] (C)	Tai Chi, 18-form Yang style	Group:1 h × 2/W × 4 Ws	NI	International POI	FRT Four-stage balance test Pinch and handgrip strength Chair-stand test ROM; wrist, elbow, and shoulder	NCI; average of five domain scores (composite memory, psychomotor speed, reaction time, complex attention, and cognitive flexibility)	NI
Li, 2018 [81] (China/Chinese)	Age 60–70 years, MCI	10	10	61.4 ± 1.84 (E) 62.1 ± 2.08 (C)	Qigong, Baduanjin	1) Adoption phase: group 60 min × 5/W × 2 Ws 2) Maintenance phase: group 60 min × 5/W × 14 Ws	Community	Usual care	CM-PPT Computerized gait analysis	Erikson flanker task More-odd shifting task 2-back task	Adjust the exercise intensity according to heart rate
Lipsitz, 2019 [82] (USA/English)	Age ≥ 60 years, healthy	93 (30:63)	87 (30:57)	75.9 ± 9.1 (E) 74.6 ± 8.6 (C)	Tai Chi, 9-form Yang style	Group intervention × 2/W and home practice 20 min × 3/W × 52 Ws *(6-month effect reported as a recommendation to end the trial by the Data and Safety Monitoring Board)*	Housing facility	Health education (monthly)	SPPB SF-12 (physical health) PASE Gait velocity	TMT: B–A	Minor musculoskeletal complaints
Chewning, 2020 [83] (USA/English)	Age ≥ 65 years, Fall risk	94 (18:76)	103 (13:90)	75.0 ± 7.4 (E) 72.8 ± 7.0 (C)	Tai Chi, Yang style	Group: 1.5 h × 2/W × 6 Ws and home-practice coaching	Community	No treatment	TUG test Chair-stand test Four-stage balance test	TMT B	NI
Zheng, 2020 [84] (China/English)	Age 45–75 years, stroke survivors with MCI	24 (19:5)	24 (22:2)	61.6 ± 9.2 (E) 62.8 ± 6.4 (C)	Qigong, Baduanjin	Group: 40 min × 3/W × 24 Ws	Community	No treatment	MBI	MoCA TMT: B–A AVLT TAP DSC CDT	No adverse events
Wu, 2021 [85] (China/English)	Age 50–85 years, Healthy	19 (4:15)	19 (1:18)	63.6 ± 4.0 (E) 63.2 ± 4.4 (C)	Tai Chi, 24-form Yang style	Group: 60 min × tri-weekly × 12 Ws	NI	Telephone consultation (every other week)	Knee extensor strength, 6MWT	IED	NI
Jiayuan, 2022 [86] (China,/English)	Age ≥ 65 years, Prefrail and frail	31 (11:18)	30 (13:17)	71.7 ± 3.9 (E) 70.8 ± 4.2 (C)	Tai Chi, 24-form Yang style	1) 1^st^ stage: Group 60 min × 2/W × 3 Ms 2) 2^nd^ stage: Individual practice 60 min × 2/W × 3 Ms	Community	Booklet about mindfulness skill	SPPB TUG test Chair-stand test	MMSE	NI
Lavretsky, 2022 [87] (USA/English)	Age ≥ 60 years, geriatric depression	89 (27:62)	89 (22:67)	69.2 ± 6.9 (E) 69.4 ± 6.2 (C)	Tai Chi	1) Group: 60 min × 1/W × 6 Ws and home practice for 20 min/day using handouts 2) Virtual: 60 min × 1/W × 6 Ws and home practice for 20 min/day using handouts	Community	Health education and wellness training	SF-36	CVLT ROCFT TMT: B–A Stroop interference COWAT Animal fluency Boston Naming Test	No adverse events

*Abbreviations: M* Male, *F* Female, *E* Experimental group, *C* Control group, *AC* Active control group, *UC* Usual care group, *TCQ* Tai Chi and Qigong, *M* Month, *W* Week, *NI* No information, *MCI* Mild cognitive impairment, *6MWT* 6-min walk test, *ADA* Alzheimer’s Disease Assessment Scale, *AVLT* Rey Auditory Verbal Learning Test, *BBS* Berg Balance Scale, *CDT* Clock-drawing task, *CI* Cerebral infarction, *CM-PPT* Chinese Mini Physical Performance Test, *COWAT* Controlled Oral Word Association Test, *CVLT* California Verbal Learning Test, *DSC* Digit symbol coding, *FRT* Functional reach test, *IED* Intra-Extra Dimensional Set Shift, *LOTCA-G* Loewenstein Occupational Therapy Cognitive Assessment–Geriatric, *MBI* Modified Barthel Index, *MCI* Mild cognitive impairment, *MMSE* Mini Mental Status Examination, *MoCA* Montreal Cognitive Assessment, *NCI* Neurocognition index, *OA* Osteoarthritis, *OLS* One-leg-standing test, *PASE* Physical Activity Scale for the Elderly, *PPA* Physiological profile assessment, *ROCFT* Rey Complex Figure Test, *ROM* Range of motion, *SF-12* 12-Item Short-Form Health Survey, *SF-36* 36-Item Short-Form Health Survey, *SPPB* Short Physical Performance Battery, *TAP* Test of Attention Performance, *TMT* Trail-Making Test, *TUG* Timed Up-and-Go Test, *WOMAC* Western Ontario and McMaster Universities Osteoarthritis Index

^a^ Analyzed for each control group;

^b^ Data are mean ± standard-deviation [range] values

The physical-function outcomes were activities of daily living (ADLs), dynamic balance, static balance, walking ability, muscle strength, flexibility, and physical health. ADLs were measured using the modified Barthel Index, and dynamic balance was measured using the Berg Balance Scale, Tinetti balance test, or functional reach test. Walking ability was measured using the Timed Up-and-Go test, computerized gait analysis, 6-min walk tests, gait velocity, Tinetti gait test, and 5-m or 10-m walking speed test. Static balance was measured using the one-leg-standing test or four-stage balance test. Muscle strength was measured using arm curls, chair-stand test, sit-to-stand test, pinch or handgrip strength, or knee extensor strength. Flexibility was measured using a back-scratch test, sit-and-reach test, or range of motion. Physical health was measured using the physical health domains of the 36- or 12-Item Short-Form Health Survey, or the physical functioning domains of the Western Ontario and McMaster Universities Osteoarthritis Index, Physiological Profile Assessment, Short Physical Performance Battery, Physical Activity Scale for the Elderly, or Chinese Mini Physical Performance Test.

The cognitive-function outcomes were cognitive performance, attention/executive function, memory, and linguistic competence. Cognitive performance was measured using the Mini-Mental State Examination (MMSE), Montreal Cognitive Assessment (MoCA), Loewenstein Occupational Therapy Cognitive Assessment–Geriatric (LOTCA-G), or the cognitive subscale of the Alzheimer’s Disease Assessment Scale (ADA). Attention/executive functions were measured using the animal-naming test, digit span (forward/backward), visual span, Trail-Making Test, Test of Attentional Performance, digit symbol coding, clock-drawing task, Eriksen flanker task, More-odd shifting task, or Stroop test. Memory was measured using the California Verbal Learning Test, Rey Auditory Verbal Learning Test, 2-back task, Intra-Extra Dimensional Set Shift, or delayed recall (e.g., Wechsler Memory Scale or Rey Complex Figure Test). Linguistic competence was measured using verbal fluency, the Controlled Oral Word Association Test, the animal-naming test (fluency), or Boston Naming Test.

### Safety monitoring

Safety monitoring was reported in 10 of the 17 studies (58.8%). There were no adverse events in eight studies and minor musculoskeletal complaints in one. In another study, exercise intensity was adjusted according to the monitored heart rate. There were no safety monitoring reports in 7 studies.

### Meta-analysis: synthesis of results

#### Effects of TCQ on cognitive function

**Fig. 3:**
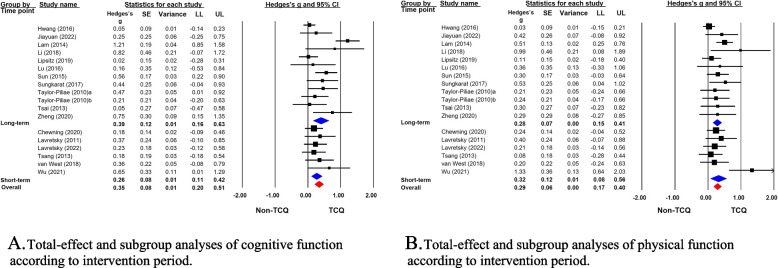
Forest plots of the effects of TCQ on cognitive and physical functions. SE, standard error; CI, confidence interval; LL, lower limit; UL, upper limit

**Fig. 4:**
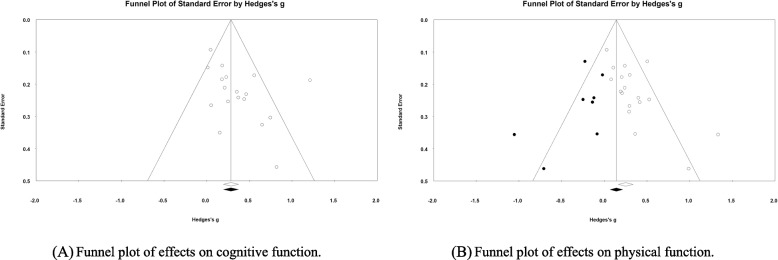
Publication bias in the included studies

The meta-analysis of the 17 studies using the random-effects model indicated that TCQ had a small effect size in improving cognitive function (Hedges’ g = 0.35, 95% CI = 0.20 to 0.51) (Fig. 3A). Potential heterogeneity was indicated by a 95% PI of − 0.21 to 0.92. There was a risk of publication bias according to the funnel plots and Egger’s regression test (*p* = 0.049) (Fig. 4A).

A subgroup comparison was conducted between long-term (k = 11) and short-term (k = 6) interventions. The effect sizes of TCQ on cognitive function were indicated by Hedges’ g values of 0.39 (95% CI = 0.16 to 0.63; 95% PI =  − 0.40 to 1.19) and 0.26 (95% CI = 0.11 to 0.42; 95% PI = 0.04 to 0.49) for long- and short-term interventions, respectively. No significant differences were found between the effect sizes according to the intervention duration (Q = 0.82, *p* = 0.364) (Fig. 3A). The funnel plot of the overall effect size for cognitive function was symmetrical, and there was no publication bias for long-term interventions according to Egger’s regression test (*p* = 0.152). However, the funnel plot for short-term interventions was asymmetrical, so publication bias may have been present.

#### Effects of TCQ on physical function

The meta-analysis of the 17 studies using the random-effects model indicated that TCQ had a small effect size in improving physical function (Hedges’ g = 0.29, 95% CI = 0.17 to 0.40) (Fig. 3B). Potential heterogeneity was indicated by a 95% PI of − 0.02 to 0.60. Publication bias was suspected according to the funnel plots and Egger’s regression test (*p* = 0.004) (Fig. 4B).

A subgroup comparison was conducted between long-term (k = 11) and short-term (k = 7) interventions. The effect sizes of TCQ on physical function were indicated by Hedges’ g values of 0.28 (95% CI = 0.15 to 0.41; 95% PI =  − 0.02 to 0.57) and 0.32 (95% CI = 0.08 to 0.56; 95% PI =  − 0.37 to 1.01) for long- and short-term interventions, respectively. No significant differences were found between the effect sizes according to the intervention duration (Q = 0.081, *p* = 0.776) (Fig. 3B). The funnel plot of the overall effect size for physical function was symmetrical, but Egger’ s regression test indicated publication bias for the long-term interventions (*p* = 0.042). The funnel plot for short-term interventions was asymmetrical, suggesting publication bias.

#### Meta-regression analysis of the effect of TCQ on cognitive function

Since TCQ had significant effects on cognitive and physical functions, we used meta-regression to explore the effect size of TCQ on cognitive function in association with the level of physical functions. The regression model was significant (Q = 9.06, *p* = 0.002), indicating that physical function significantly impacts the effects of TCQ on cognitive function. The variance that was explained by the model was 0.035 within the total of 0.056, indicating that 63% of the heterogeneity was explained by the model that used physical function as a moderator (Table 2). This confirmed that changes in physical function were associated with changes in cognitive function. The goodness-of-fit test also addressed whether there was heterogeneity that could not be explained by physical function as a covariate. Q_resid_ was used to estimate the variance of the unexplained heterogeneity. The variance that was not explained by the model was 0.021, and the goodness-of-fit test indicated that the unexplained variance was not zero (Q = 26.04, df = 16, *p* = 0.05). In conclusion, the effects of TCQ on cognitive function remained significant when controlling for the effect of physical function in this model.

**Table 2:** Meta regression random-effects model: test of the model

Simultabneous test that all coefiicients (excluding intercept) are zero Q_model_ = 9.06, df = 1, *p* = 0.0026
Goodness of fit: Test that unexplained variance is zero T^2^ = 0.0208, Tau = 0.1442, I^2^ = 38.56%, Q_resid_ = 30.766, df = 16, *p* = 0.0534
Proportion of total between-study variance explained by the model R^2^ = T^2^_explained_/T^2^_total_ = 0.0352/0.0560 = 0.63

## Discussion

This study evaluated the effect of TCQ on cognitive function with physical function as a covariate among older adults with cognitive impairments. The obtained evidence supported the beneficial effects of TCQ on cognitive functions, including global, memory, and executive functions in individuals with or without cognitive impairments [16, 88]. Tai Chi or Qigong was administered for at least 12 weeks and was considered to be a promising alternative mind–body intervention for older adults with MCI [61]. The present review included 17 studies (14 Tai Chi and 3 Qigong interventions) with a mean duration of 19.7 weeks (range = 4–52 weeks). The meta-analysis with the random-effects model also confirmed the significant effects of TCQ on cognitive function for short-term (≤ 12 weeks) and long-term interventions. The difference in effect sizes between the intervention durations was also not significant in our study.

A recent meta-analysis of 14 studies on the effect of aerobic or resistance exercise on older adults with MCI confirmed the beneficial effects of TCQ, including improvement in cognitive function, and in the grip strength of physical function [13]. The association between cognitive and physical functions under the impact of exercise training indicates that exercise-induced improvements in physical function are associated with improved cognitive function [59]. One of the common mechanisms underlying exercise-induced benefits in cognitive function is related to neuroplasticity being facilitated in certain brain structures by physical activity [23]. Rogers and colleagues [24] conducted a 4-year prospective longitudinal study to examine the effects of different physical activity levels on cerebral perfusion and found that physically inactive, retired older adults presented significant declines in cerebral blood flow throughout four years of follow-up compared with those who regularly engaged in physical activity. It is well established that physical activity can increase circulating levels of a BDNF with cognitively beneficial properties. However, the type, intensity, and duration of physical exercise that would induce this mechanism are still under debate [89].

The typical features of TCQ as a mind–body exercise are physical and cognitive-stimulating activities [90]. A previous systematic review based on seven randomized trials proposed that changes in cognitive function during TCQ training may be associated with structural and functional changes in the cortex related to cognition [88]. In addition to the common mechanism through conventional exercise, TCQ as a mind–body exercise could also induce improvements in brain neuroplasticity through its motor complexity and multiple components combined with meditation training and relaxation practice [91, 92]. The aerobic exercise components of TCQ can delay age-related brain atrophy and improve cerebral blood circulation [23, 24], while the tranquility of mindfulness and Qigong breathing can improve attentional focus and executive function [34, 36]. A randomized trial compared the effects of Tai Chi with conventional exercise on cognitive function, and found a significant improvement in older adults from the Tai Chi group at 12 weeks. Both groups achieved clinically relevant improvements at 24 weeks [91].

Given that exercise-induced benefits on physical and cognitive functions are interrelated, TCQ intervention has been hypothesized as a beneficial mind–body exercise that may affect cognitive function either directly or indirectly via improving physical function. In order to confirm the direct pathway of TCQ on cognitive function, the present study used meta-regression analysis to explore further the effect of TCQ on cognitive function with physical function as a moderator. This meta-regression was conducted on the 17 studies in the review, since the recommended minimum number of studies for meta-regression analysis is 10 [63]. Including physical function as a moderator in the model resulted in explaining 63% of the variance in the true effects of TCQ. In other words, physical function as a moderator was significantly related to the effect size from TCQ. Although no previous study has examined the effect of TCQ on cognitive function using meta-regression, the available evidence supports the effect of TCQ on physical function in various populations including older adults with or without chronic conditions [93]. The next step was to explore whether the model with physical function as a moderator explained all of the variance in the effect size. The unexplained variance was still significant, leading to the conclusion that TCQ still have significant effects on cognitive function even after controlling for the effects from physical function.

The meta-regression suggested that TCQ acts beneficially on cognitive function via various mechanisms, not just in its aerobic components but also in the cognitive process of memorizing movements and paying attention to meditation. Therefore, further studies are warranted to explore the underlying mechanisms of TCQ-induced health benefits on physical and cognitive functions. This might reveal the rationale for exercise interventions such as TCQ to improve quality of life by slowing down the decline in cognitive function among those with cognitive impairment.

Some strengths and limitations should be considered when interpreting the present results. The strengths of the study were that we included 17 randomized trials searched from databases in 3 different languages with both cognitive and physical functions as outcomes, which enabled us to conduct the meta-regression analysis. However, the heterogeneity of the studies was also noted due to the range of older adults with varying health and cognitive statuses. The types of Tai Chi or Qigong, although all based on theoretical principles of traditional Chinese medicine, were different in intervention durations or intensity. Moreover, physical and cognitive functions were assessed using various measurement methods. Considering the heterogeneity of the studies, we used random-effect model for the main analysis and subgroup analysis on intervention duration with more than 5 studies. The sensitivity analysis with excluding outliers was also conducted to confirm the effects of TCQ.

## Conclusions

The findings of this review suggest that TCQ effectively improves cognitive and physical functions among older adults with or without cognitive impairment when implemented as both short-term and long-term programs. The effect of TCQ on cognitive function remained significant after taking into account the significant effects of physical function as a moderator. The findings imply the potential health benefits of TCQ by promoting cognitive function in older adults directly and indirectly through enhancing physical function.

## Supplementary Information

**Additional file 1: Appendix.** Search Strategy; Pubmed.

## Data Availability

The datasets used and analyzed during the current study are available from the corresponding author upon reasonable request.

## Abbreviations

TCQ: Tai Chi and Qigong
BDNF: Brain-derived neurotrophic factor
MCI: Mild cognitive impairment
RoB: Risk of bias
PI: Prediction interval
