# Supplementary material for: Effects of Tai Chi and Qigong on cognitive and physical functions in older adults: systematic review, meta-analysis, and meta-regression of randomized clinical trials
Source: BMC Geriatr. 2023 Jun 6;23:352. doi: 10.1186/s12877-023-04070-2 (PMC10242998; doi:10.1186/s12877-023-04070-2)
Supplement: Supplementary file 1 — Additional file 1: Appendix. Search Strategy; Pubmed. [file 12877_2023_4070_MOESM1_ESM.docx]

Appendix. Search Strategy; Pubmed

| Search Number | | Query | Results |
| --- | --- | --- | --- |
| #1 | #1 | "Tai-ji"[MeSH Terms] OR "Qigong"[MeSH Terms] OR "tai chi"[Title/Abstract] OR "taiji"[Title/Abstract] OR "taichi"[Title/Abstract] OR "Tai-ji"[Title/Abstract] OR "t ai chi"[Title/Abstract] OR "Tai-ji"[Title/Abstract] OR "Taijiquan"[Title/Abstract] OR "Taichichuan"[Title/Abstract] OR "Qigong"[Title/Abstract] OR "chi kung"[Title/Abstract] OR "qi gong"[Title/Abstract] OR "martial art"[Title/Abstract] | 3,095 |
| #2 | #2 | "random allocation"[MeSH Terms] OR "clinical trials as topic"[MeSH Terms] OR "controlled clinical trials as topic"[MeSH Terms] OR "random*"[Title/Abstract] OR "RCT"[Title/Abstract] OR "controlled clinical"[Title/Abstract] OR "clinical trial*"[Title/Abstract] | 1,790,163 |
| #3 | #3 | "mental processes"[MeSH Terms] OR "neuropsychological tests"[MeSH Terms] OR "cognit*"[Title/Abstract] OR "memor*"[Title/Abstract] OR "recogni*"[Title/Abstract] OR "think*"[Title/Abstract] OR "attention"[Title/Abstract] OR "decision making"[Title/Abstract] OR "mental"[Title/Abstract] OR "learn*"[Title/Abstract] OR "problem solving"[Title/Abstract] OR "aware*"[Title/Abstract] OR "anticipat*"[Title/Abstract] OR "executive function"[Title/Abstract] | 3,652,869 |
| #4 | #1 AND #2 | ("Tai-ji"[MeSH Terms] OR "Qigong"[MeSH Terms] OR "tai chi"[Title/Abstract] OR "taiji"[Title/Abstract] OR "taichi"[Title/Abstract] OR "Tai-ji"[Title/Abstract] OR "t ai chi"[Title/Abstract] OR "Tai-ji"[Title/Abstract] OR "Taijiquan"[Title/Abstract] OR "Taichichuan"[Title/Abstract] OR "Qigong"[Title/Abstract] OR "chi kung"[Title/Abstract] OR "qi gong"[Title/Abstract] OR "martial art"[Title/Abstract]) AND ("random allocation"[MeSH Terms] OR "clinical trials as topic"[MeSH Terms] OR "controlled clinical trials as topic"[MeSH Terms] OR "random*"[Title/Abstract] OR "RCT"[Title/Abstract] OR "controlled clinical"[Title/Abstract] OR "clinical trial*"[Title/Abstract]) | 1,374 |
| #5 | #3 AND #4 | ("Tai-ji"[MeSH Terms] OR "Qigong"[MeSH Terms] OR "tai chi"[Title/Abstract] OR "taiji"[Title/Abstract] OR "taichi"[Title/Abstract] OR "Tai-ji"[Title/Abstract] OR "t ai chi"[Title/Abstract] OR "Tai-ji"[Title/Abstract] OR "Taijiquan"[Title/Abstract] OR "Taichichuan"[Title/Abstract] OR "Qigong"[Title/Abstract] OR "chi kung"[Title/Abstract] OR "qi gong"[Title/Abstract] OR "martial art"[Title/Abstract]) AND ("random allocation"[MeSH Terms] OR "clinical trials as topic"[MeSH Terms] OR "controlled clinical trials as topic"[MeSH Terms] OR "random*"[Title/Abstract] OR "RCT"[Title/Abstract] OR "controlled clinical"[Title/Abstract] OR "clinical trial*"[Title/Abstract]) AND ("mental processes"[MeSH Terms] OR "neuropsychological tests"[MeSH Terms] OR "cognit*"[Title/Abstract] OR "memor*"[Title/Abstract] OR "recogni*"[Title/Abstract] OR "think*"[Title/Abstract] OR "attention"[Title/Abstract] OR "decision making"[Title/Abstract] OR "mental"[Title/Abstract] OR "learn*"[Title/Abstract] OR "problem solving"[Title/Abstract] OR "aware*"[Title/Abstract] OR "anticipat*"[Title/Abstract] OR "executive function"[Title/Abstract]) | 503 |
